# Supplementary figures and images for: Comparison of rule- and ordinary differential equation-based dynamic model of DARPP-32 signalling network
Source: PeerJ. 2022 Dec 15;10:e14516. doi: 10.7717/peerj.14516 (PMC9760030; doi:10.7717/peerj.14516)

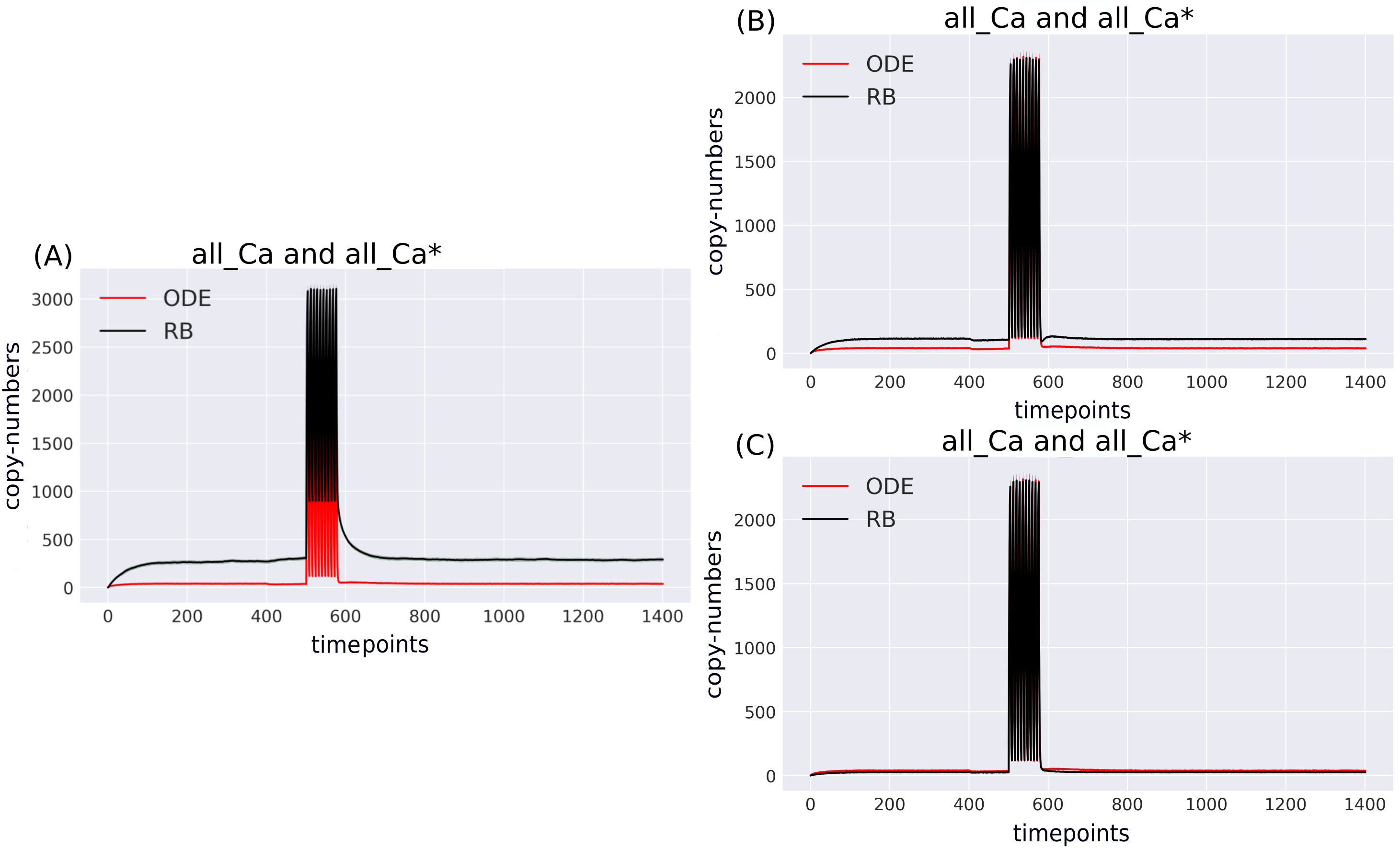

Supplement: Supplemental Information 1 — Comparison of variable compositions of molecular species containing Ca2+ ions tracked in the system in both models with (A) unaltered observables; (B) all molecular species containing Ca2+ ions selected by names to match the original model and summed to obtain a single trace, where 13 molecular species in the ODE model are represented by 18 species in the RB model; (C) 13 molecular species of ODE model matched to 13 of RB model, where only 1 of 6 molecular species of inactive PP2B was selected. In comparison to the unaltered species composition (A), the result shows that discrepancy between the ODE and RB observable trajectories have diminished (B,C). [file peerj-10-14516-s001.png]

copy-numbers

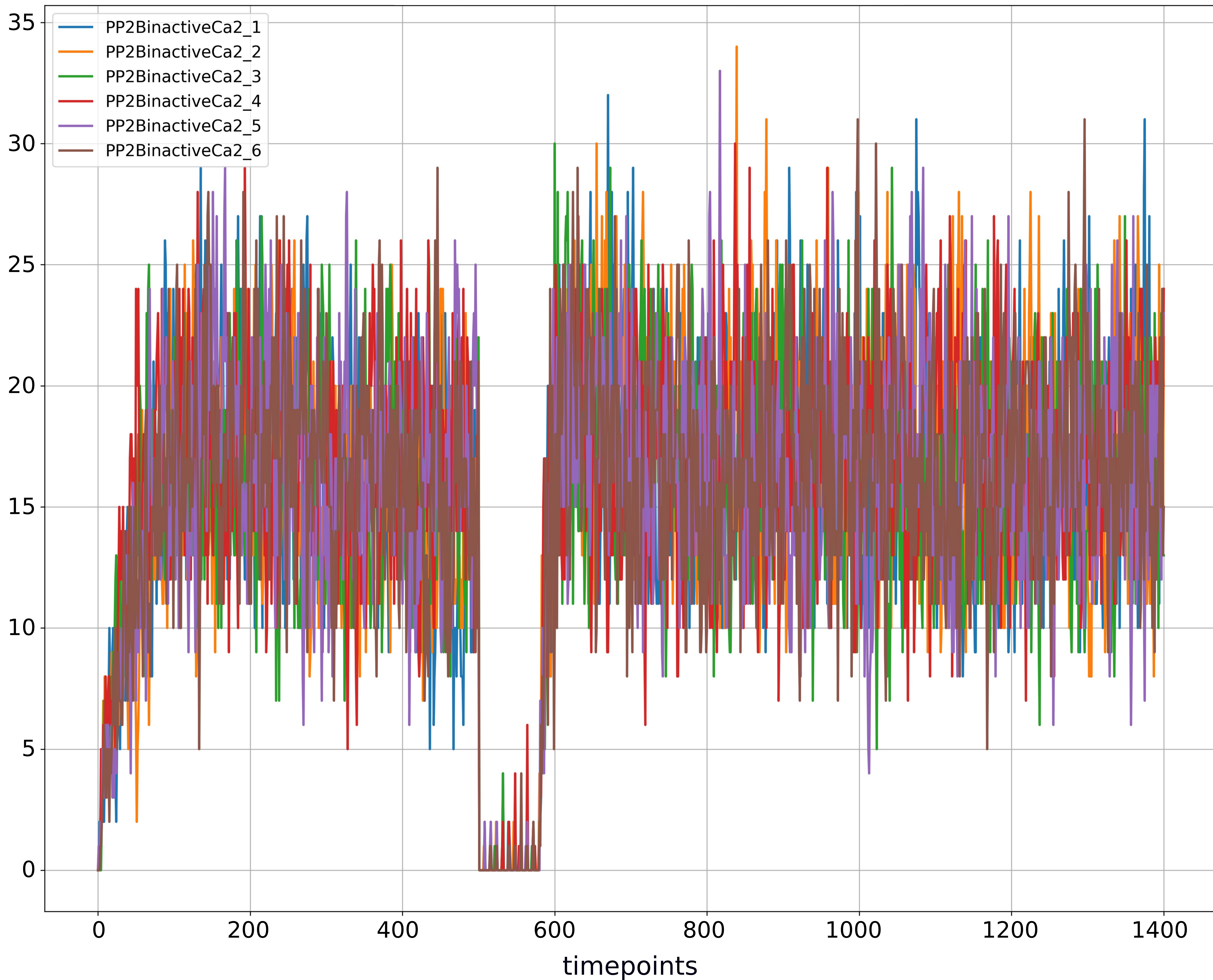

Supplement: Supplemental Information 2 — Half-active PP2B is a complex composed of PP2B and two Ca2+ ions. Simulation of the RB model generates six different molecular species representing this complex due to combinatorial binding of Ca2+ ions to four identical PP2B sites. The graph shows the superimposed trajectories of these six variants of half-active PP2B. None of these six trajectories is distinguished from the others by either the pattern of dynamics or the average abundance level. [file peerj-10-14516-s002.pdf]

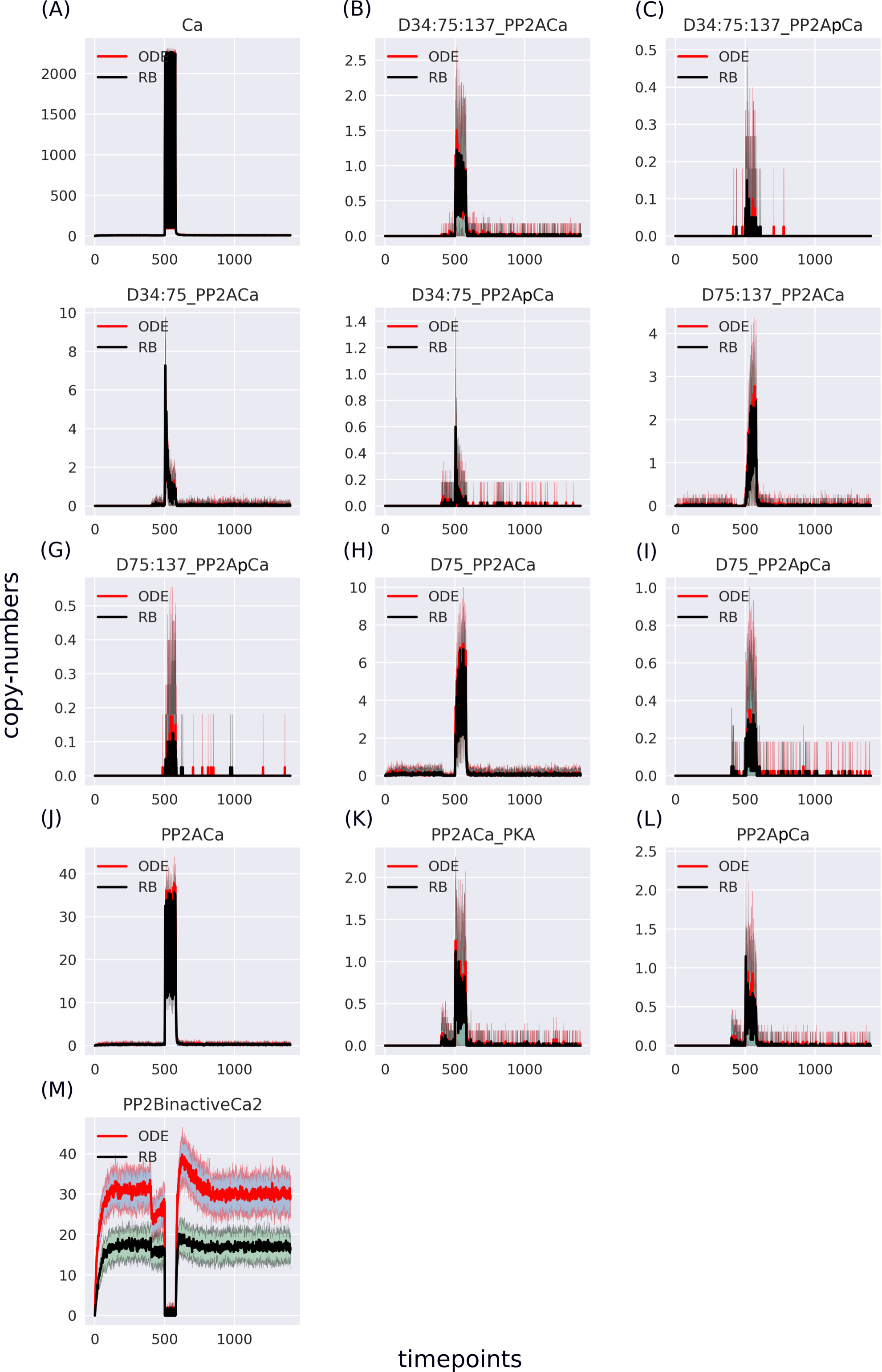

Supplement: Supplemental Information 3 — Comparison of separated Ca2+-containing molecular species selected as in the ODE model. The “PP2BinactiveCa2” trajectory in the RB model was obtained by selecting one of 6 entities representing, among others, the inactive form of PP2B in the RB model. There is still a discrepancy between the models, but the trajectory is lower for the RB model. [file peerj-10-14516-s003.pdf]

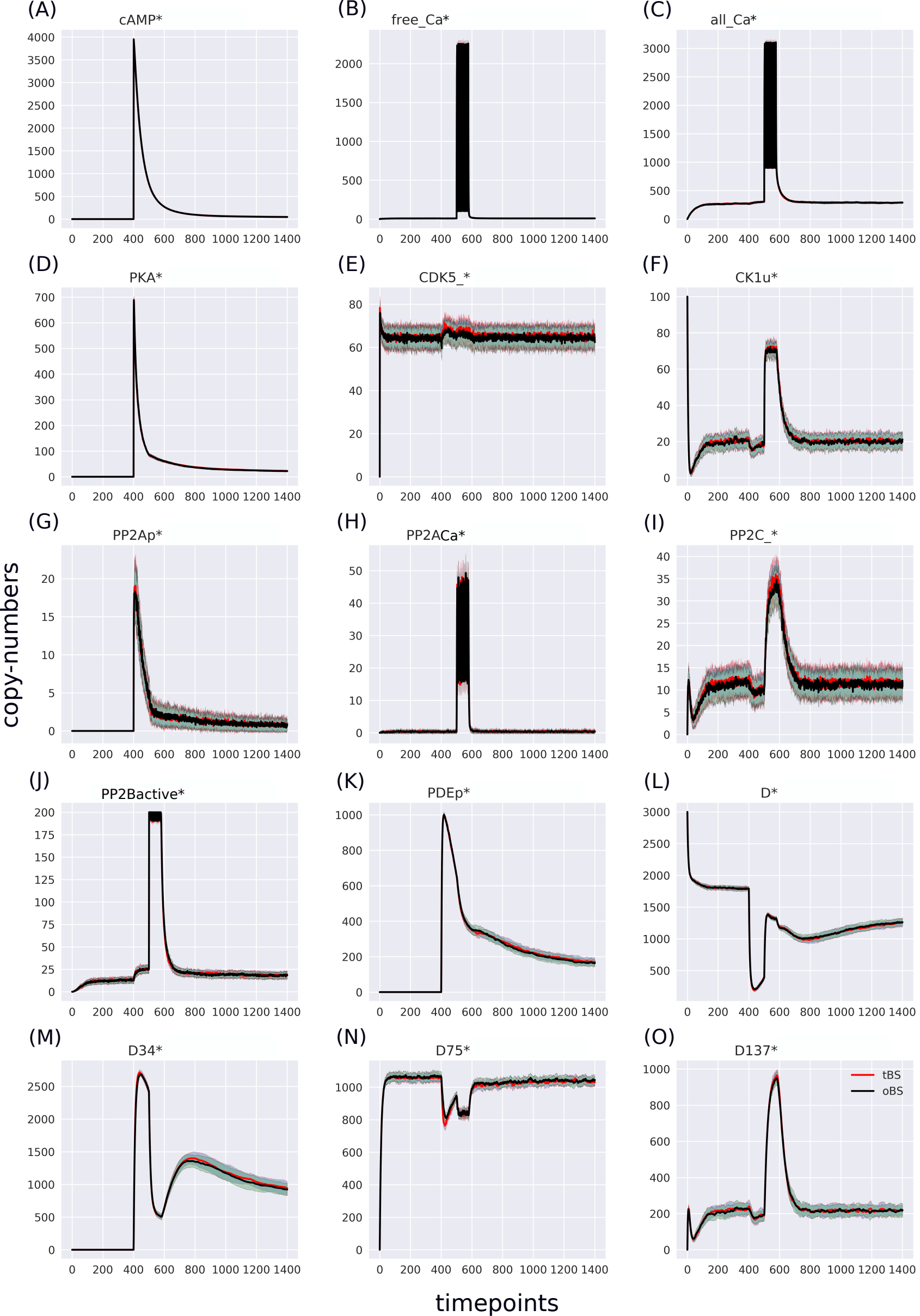

Supplement: Supplemental Information 4 — Comparison of two variants of the RB model in which the agent representing DARPP-32 had one binding site (oBS, red trace) and three binding sites (tBS, black trace). The superimposed trajectories of the respective agents indicate that the model trajectories were not affected by this modification. [file peerj-10-14516-s004.pdf]

(A)

Number of set species between 0s and 699s

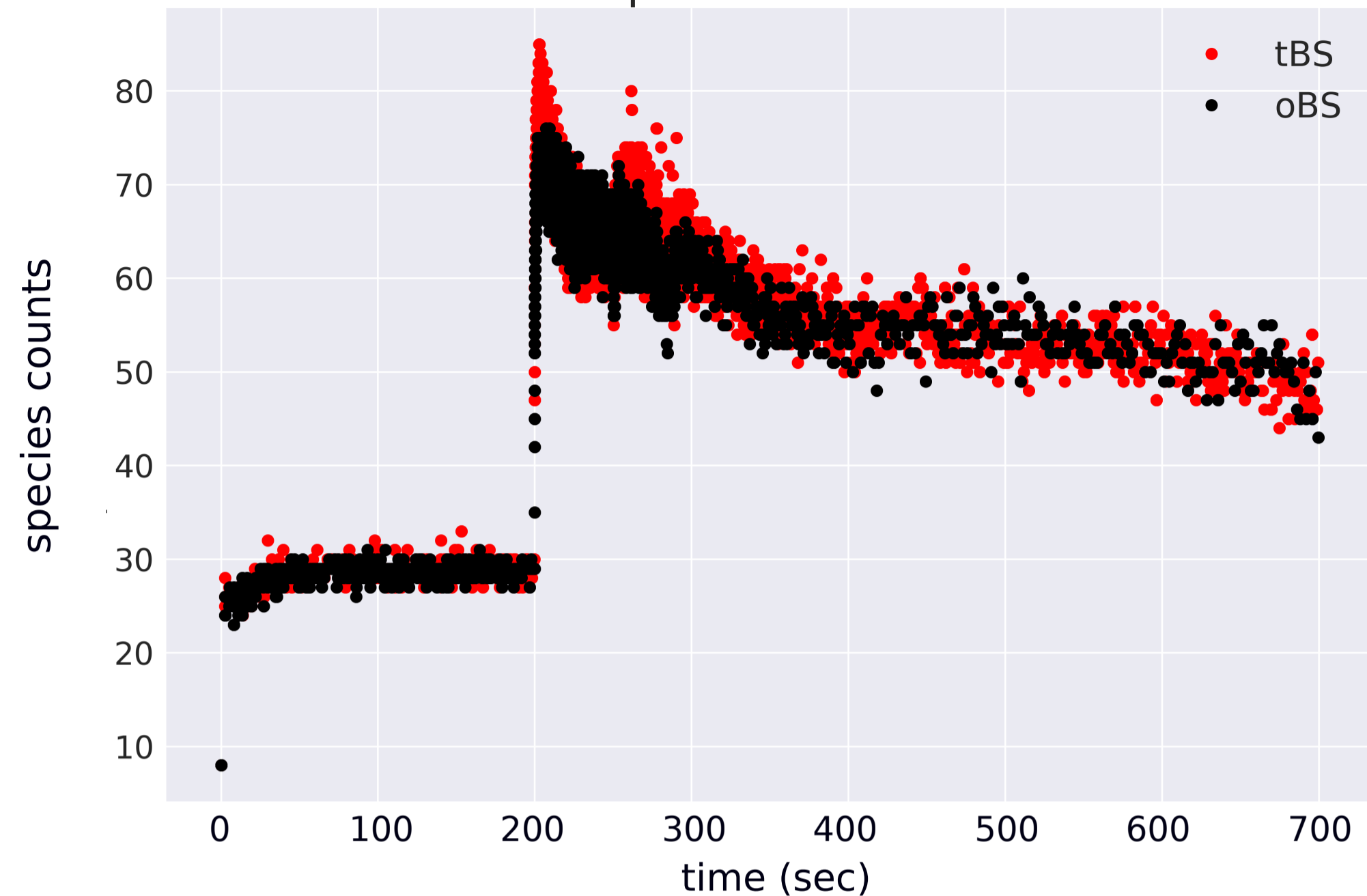

(B)

Ca<sup>2+</sup> and cAMP stimuli

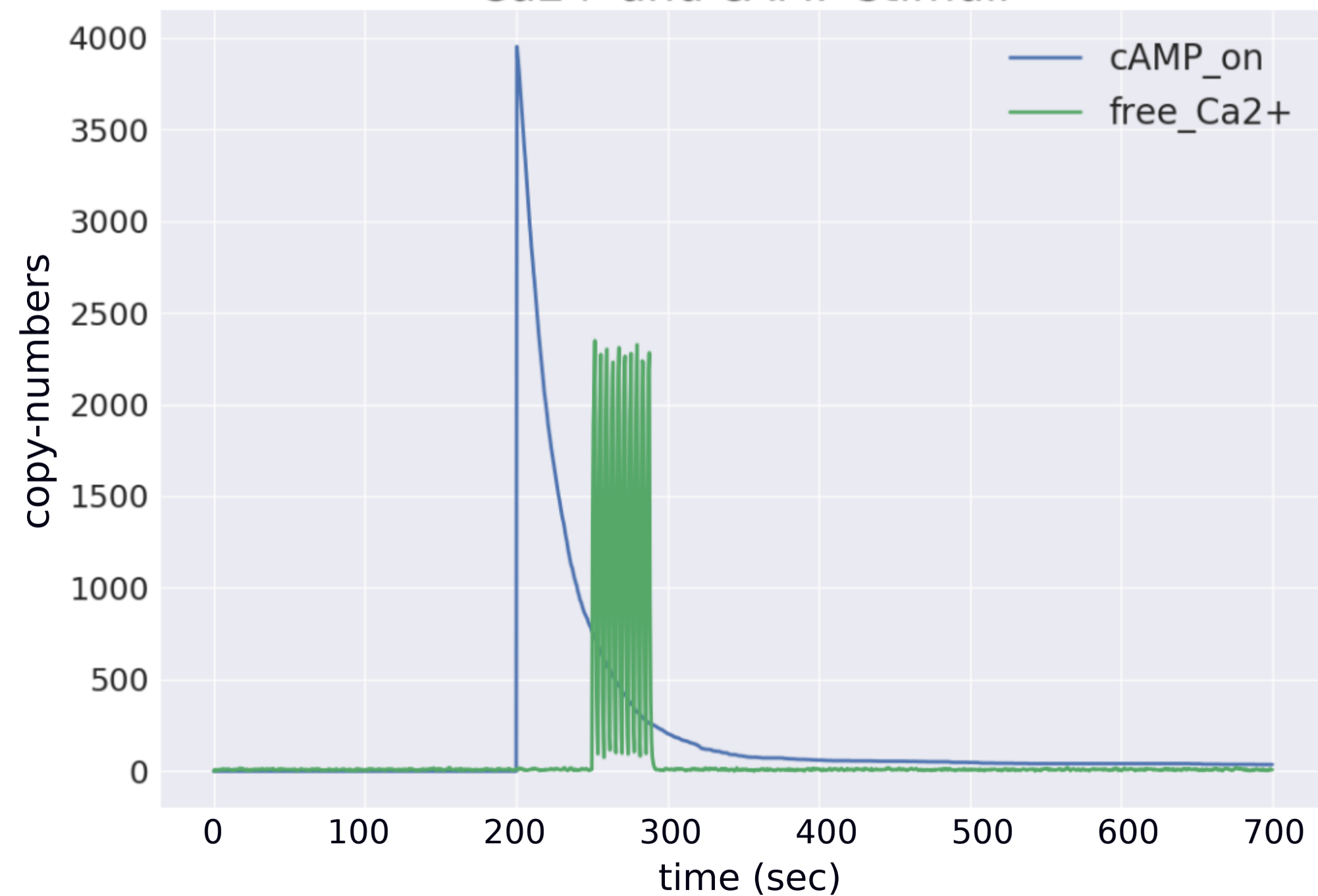

Supplement: Supplemental Information 5 — Overlay of changes in the number of species over time for two models in which DARPP-32 can bind at one site (oBS) and three sites (tBS) (A). The size of the species set is similar in both model variants. The change in the number of unique species is consistent with the stimulus trajectory (B). The dynamics of complex formation are dictated by the pattern of stimulus input, as the largest differences between oBS and tBS occur during stimulus application. [file peerj-10-14516-s005.pdf]
