# Supplementary material for: Comparison of rule- and ordinary differential equation-based dynamic model of DARPP-32 signalling network
Source: PeerJ. 2022 Dec 15;10:e14516. doi: 10.7717/peerj.14516 (PMC9760030; doi:10.7717/peerj.14516)
Supplement: Supplemental Information 6 — Names of RB observables and corresponding names of ODE observables with definitions. To obtain observable ODEs, the time series of the corresponding molecular species are summed based on their names. [file peerj-10-14516-s006.pdf]

RB	ODE	Definition
cAMP*	cAMP	cAMP binding unspecified
free_Ca*	free_Ca	Ca^{2+} unbound
all_Ca*	all_Ca	Ca^{2+} binding unspecified
PKA*	PKA	PKA binding unspecified
CDK5_*	_CDK5	CDK5 bound
CK1u*	CK1u	CK1 unphosphorylated, binding unspecified
PP2Ap*	PP2Ap	PP2A phosphorylated, all bindings unspecified
PP2ACa*	PP2ACa	PP2A bound to Ca^{2+}, phosphorylation and other bindings unspecified
PP2C_*	_PP2C	PP2C bound
PP2Bactive*	PP2Bactive	PP2B active, binding unspecified
PDEp*	PDEp	PDE phosphorylated, binding unspecified
D*	D	DARPP-32 unphosphorylated at all sites, binding unspecified
D34*	D34	DARPP-32 phoshophorylated at Thr34 with unspecified binding, other sites' internal states and binding unspecified
D75*	D75	DARPP-32 phoshophorylated at Thr75 with unspecified binding, other sites' internal states and binding unspecified
D137*	D137	DARPP-32 phoshophorylated at Ser137 with unspecified binding, other sites' internal states and binding unspecified
