# Supplementary material for: Comparison of rule- and ordinary differential equation-based dynamic model of DARPP-32 signalling network
Source: PeerJ. 2022 Dec 15;10:e14516. doi: 10.7717/peerj.14516 (PMC9760030; doi:10.7717/peerj.14516)
Supplement: Supplemental Information 7 [file peerj-10-14516-s007.pdf]

SUPPLEMENTARY MATERIALS:

COMPARISON OF RULE- AND ORDINARY DIFFERENTIAL EQUATION-BASED DYNAMIC MODEL OF DARPP-32 SIGNALLING NETWORK

1 AUTOMATED TRANSLATION OF ODE MODEL WITH ATOMIZER

BioNetGen (BNG) is a member of the family of rule-based formalisms that most closely resemble Kappa. The BNG framework is supported by the automated translation of SBML into BNGL format by Atomizer (Tapia and Faeder, 2013). Atomizer defines molecular bond structures, implicit in reaction-based models, through the use of molecular species naming conventions and reaction stoichiometry. This method could potentially offer an easy way to derive an RB model from the original ODE. The performance of the Atomizer has been tested on the Fernandez et al. (2006) model to compare the results of this automatic translation with a manual translation of the model. The automatic translation was successful, but simulation of the resulting model resulted in errors, regardless of using all available model simulation options. The standard error output reported many instances of conflicting definitions and inconsistent naming. In notational terms, the complexity and redundancy of the model coding appeared not to be designed to be edited by a human and therefore it was difficult to assess correctness without simulating the model. The generated model was examined in terms of agent and rule definitions. Examination of the rules revealed fully contextualised reaction instances (Code 3). All combinations of DARPP-32 states were represented as separate species (Code 1, l.2 and l.3).

These results supported the need for a manual translation of the ODE model into Kappa.

Code 1. Example of agents in BNGL generated with Atomizer

```
1 CDK5(d,d137,d34,d34_137)
2 D75(ck1,pka,pp2)
3 D(cdk5,ck1,pka)
```

Code 2. Example of agent manually formulated in Kappa

```
%agent: CK1(pSite~u~p)
%agent: D(s, thr34~u~p, ser137~u~p, thr75~u~p)
```

Code 3. Example of rules in BNGL for a two-step phosphorylation generated with Atomizer

```
von1: D(cdk5,ck1,pka)@Spine + CDK5(d,d137,d34,d34_137)@Spine -> CDK5(d!2
    ,d137,d34,d34_137)@Spine.D(cdk5!2,ck1,pka)@Spine functionRate0()

voff1: CDK5(d!2,d137,d34,d34_137)@Spine.D(cdk5!2,ck1,pka)@Spine -> D(
    cdk5,ck1,pka)@Spine + CDK5(d,d137,d34,d34_137)@Spine r2_koff1

vcat1: CDK5(d!2,d137,d34,d34_137)@Spine.D(cdk5!2,ck1,pka)@Spine -> D75(
    ck1,pka,pp2)@Spine + CDK5(d,d137,d34,d34_137)@Spine r3_kcat1
```

Code 4. Example of rules for a two-step phosphorylation manually encoded in Kappa

```
D(s, thr75~u),CDK5(a) <-> D(s!1, thr75~u),CDK5(a!1)
                                      @'kon1','koff1'
D(s!1, thr75~u),CDK5(a!1) -> D(s, thr75~p),CDK5(a) @'kcat1'
```

RB	ODE	Definition
cAMP*	cAMP	cAMP binding unspecified
free_Ca*	free_Ca	Ca^{2+} unbound
all_Ca*	all_Ca	Ca^{2+} binding unspecified
PKA*	PKA	PKA binding unspecified
CDK5_*	_CDK5	CDK5 bound
CK1u*	CK1u	CK1 unphosphorylated, binding unspecified
PP2Ap*	PP2Ap	PP2A phosphorylated, all bindings unspecified
PP2ACa*	PP2ACa	PP2A bound to Ca^{2+}, phosphorylation and other bindings unspecified
PP2C_*	_PP2C	PP2C bound
PP2Bactive*	PP2Bactive	PP2B active, binding unspecified
PDEp*	PDEp	PDE phosphorylated, binding unspecified
D*	D	DARPP-32 unphosphorylated at all sites, binding unspecified
D34*	D34	DARPP-32 phoshophorylated at Thr34 with unspecified binding, other sites' internal states and binding unspecified
D75*	D75	DARPP-32 phoshophorylated at Thr75 with unspecified binding, other sites' internal states and binding unspecified
D137*	D137	DARPP-32 phoshophorylated at Ser137 with unspecified binding, other sites' internal states and binding unspecified

Table S1. Names of RB observables and corresponding names of ODE observables with definitions. To obtain observable ODEs, the time series of the corresponding molecular species are summed based on their names.

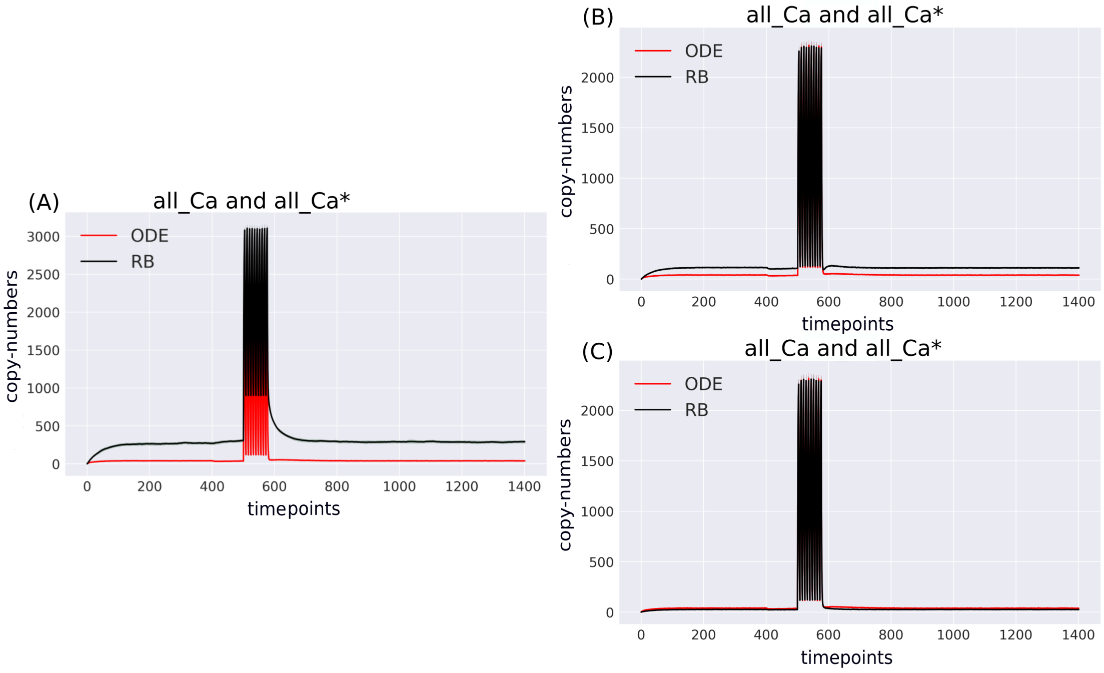

Figure S1. Comparison of variable compositions of molecular species containing Ca^{2+} ions tracked in the system in both models with (A) unaltered observables; (B) all molecular species containing Ca^{2+} ions selected by names to match the original model and summed to obtain a single trace, where 13 molecular species in the ODE model are represented by 18 species in the RB model; (C) 13 molecular species of ODE model matched to 13 of RB model, where only 1 of 6 molecular species of inactive PP2B was selected. In comparison to the unaltered species composition (A), the result shows that discrepancy between the ODE and RB observable trajectories have diminished (B,C).

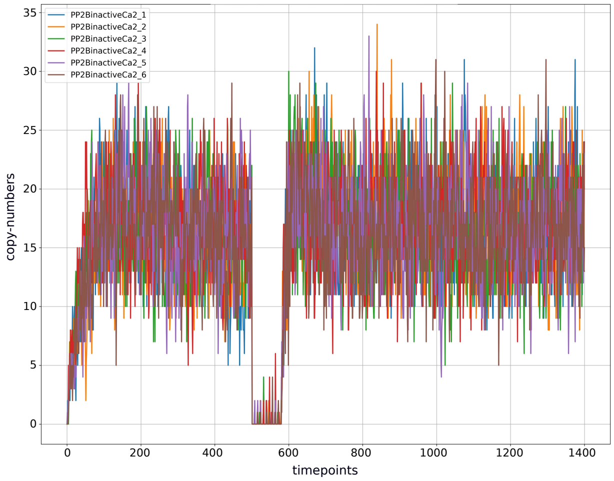

Figure S2. Half-active PP2B is a complex composed of PP2B and two Ca^{2+} ions. Simulation of the RB model generates six different molecular species representing this complex due to combinatorial binding of Ca^{2+} ions to four identical PP2B sites. The graph shows the superimposed trajectories of these six variants of half-active PP2B. None of these six trajectories is distinguished from the others by either the pattern of dynamics or the average abundance level.

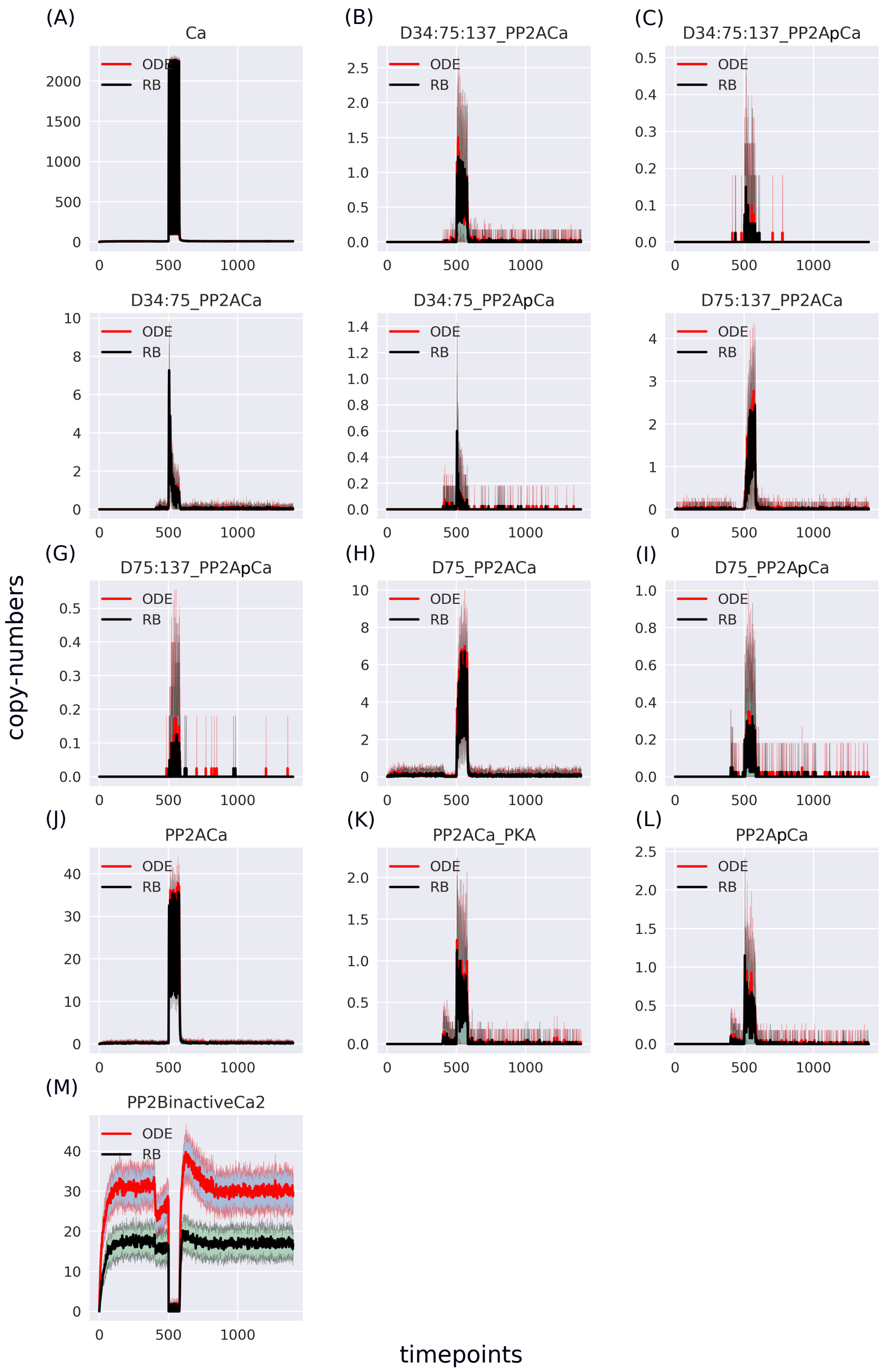

Figure S3. Comparison of separated Ca^{2+}-containing molecular species selected as in the ODE model. The “PP2BinactiveCa2” trajectory in the RB model was obtained by selecting one of 6 entities representing, among others, the inactive form of PP2B in the RB model. There is still a discrepancy between the models, but the trajectory is lower for the RB model.

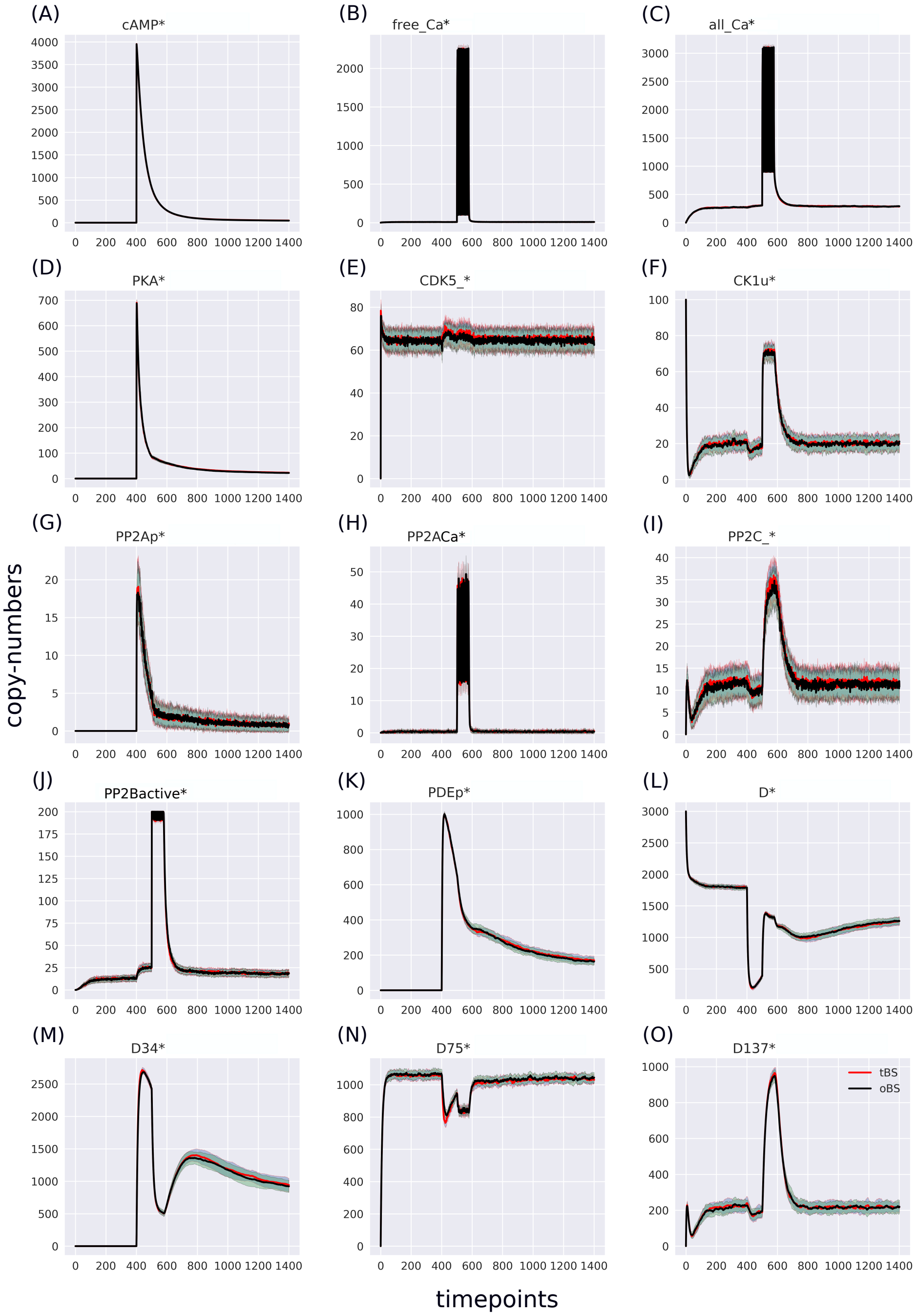

Figure S4. Comparison of two variants of the RB model in which the agent representing DARPP-32 had one binding site (oBS, red trace) and three binding sites (tBS, black trace). The superimposed trajectories of the respective agents indicate that the model trajectories were not affected by this modification.

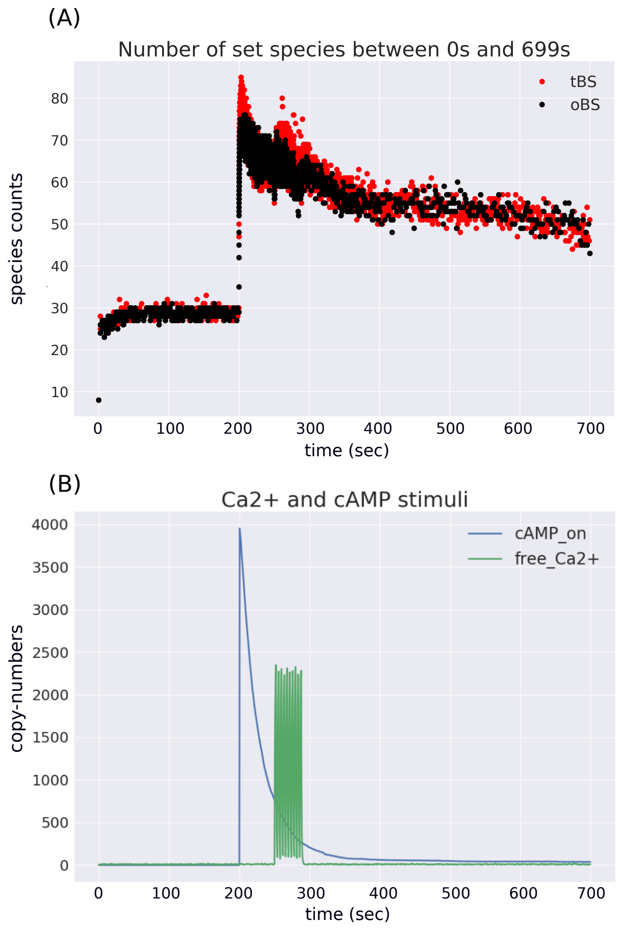

Figure S5. Overlay of changes in the number of species over time for two models in which DARPP-32 can bind at one site (oBS) and three sites (tBS) (A). The size of the species set is similar in both model variants. The change in the number of unique species is consistent with the stimulus trajectory (B). The dynamics of complex formation are dictated by the pattern of stimulus input, as the largest differences between oBS and tBS occur during stimulus application.

REFERENCES

Fernandez, É., Schiappa, R., Girault, J. A., and Le Novère, N. (2006). DARPP-32 is a robust integrator of dopamine and glutamate signals. *PLoS Computational Biology*, 2(12):1619–1633.

Tapia, J.-J. and Faeder, J. R. (2013). The Atomizer: Extracting Implicit Molecular Structure from Reaction Network Models. *Proceedings of the International Conference on Bioinformatics, Computational Biology and Biomedical Informatics (BCB'13)*, pages 726–727.
